# American Dental Association

**Published:** 1885-10

**Authors:** 


### AMERICAN DENTAL ASSOCIATION.

TWENTY-FIFTH ANNUAL MEETING, HELD AT MINNEAPOLIS, MINN., AUGUST 4, 5, 6, AND 7, 1885.

REPORTED FOR THE INDEPENDENT PRACTITIONER, BY "MRS. M. W. J."

(Continued from page 486.)

### WEDNESDAY AUGUST 5TH—MORNING SESSION.

Section II not being ready, section III was called; Literature and Nomenclature.

Dr. W. H. Atkinson read a paper entitled "Ripening and Ripeness." It was so thoroughly condensed, being almost a succession of aphorisms and definitions, that an abstract fails to convey any clear conception of its purport.

He said—ripening is the completion of process. Production is the leading forward of something which is led; this involves conscious and sub-conscious order of movement. Consciousness is independent of time and space, until arrest of movement produces sub-consciousness manifested in the senses.

Sound mental action is regular, in accordance with purpose; unsound mental action is irregular and purposeless. Perspicacity of nomenclature demands the settlement of the question of germs, the foresteps in kingdom, class, order, genus, species, and varieties. Mass changes only have been studied; molecular changes have been ignored. The first requirement is to determine the lowest form of body capable of storing heredity. If the researches thus far made are to be relied on, the smallest corpuscles are able by fission, or by producing spores, to perpetuate their kind. But little is yet known or definitely settled regarding these foresteps. In attempting to account for the origin of the atom we become lost in the cloud mass.

Atoms are unoriginated eternities. We have to begin with an assumption or postulate as a base; given this starting point, and we are led on to ether, gas, vapor, water, colloid, and solid. Assume the atom and we reach molecules, corpuscles, tissues, organs, and systems. When the molecules are arranged in regular order, the corpuscle is produced; further measures of energy fit it for arrangement into tissue; organs are built of tissues, and systems of organs. Ether is the diffusion of atomic mass; disturbance in the tension of ether generates molecular mass.

Air supports combustion, water puts it out, and the earth is formed by precipitation of the ash and dissipation of the gas. Thus the earth, the atmosphere, and the empyrean are made to appear as the observable sphere of functional activities.

Dr. W. O. Kulp read another paper on Nomenclature. He said that in all science the nomenclature was of the first importance. Each term should convey a definite idea. In dentistry there is only a general classification; each writer has his own terms. The profession demands a uniform nomenclature.

The following is a general outline of the system of nomenclature for the teeth and their surfaces, proposed by Dr. Kulp, and which has been successfully used by him in his classes:

The surfaces of the six anterior teeth, upper and lower, are labial, lingual, proximal, and occluding.

Of the bicuspids and molars—buccal, lingual, proximal and occluding.

The longitudinal surfaces (applicable to all the teeth)—grinding, middle, and cervical thirds (from cutting edge to alveolar process).

To individualize the proximal cavities, those of the central incisors are centro-proximal and latero-proximal.

Of the right and left laterals—centro-proximal and cuspo-proximal.

Of the cuspids—latero-proximal, and bicuspo-proximal.

The first bicuspids—cuspo-proximal and bicuspo-proximal, buccal cusp, and lingual cusp.

The second bicuspids—bicuspo-proximal, molo-proximal, bicuspo-fissure, buccal-cusp, and lingual-cusp.

The first upper molars—bicuspo-proximal, molo-proximal, anterior fissure, crown fissure, posterior-buccal prominence, anterior-buccal prominence, lingual prominence (the upper molars being tricuspid, as a rule).

This system being carried out to its fullest extent, every possible cavity, or any portion of each and every tooth, upper and lower, is clearly and definitely located by a single term, terse and simple.

*Dr. C. W. Spalding*—Said that the most severely felt want in the profession was the exact definition of terms, especially in the manipulative and mechanical portions of work. In the system here proposed, the terms are as brief and definite as possible, but we need a system of nomenclature which shall extend beyond the mere crown of a tooth, and beyond operative dentistry. As it is, all our terms are confused, the same word often expressing very different ideas on the one hand, with different words expressing the same idea on the other.

*Dr. A. H. Thompson*—Enquired why the terms "mesial" and "distal," brief and plain terms, now generally used, were rejected.

*Dr. Kulp*—Because the words mesial and distal convey either no meaning or a false one, to one who has only an ordinary primary education, as is too often the case with our students, while a term derived from the names of the teeth involved defines itself.

The term distal, implying most distant, conveys a false impression when applied to a cavity of the anterior teeth, indicating the palatal surface.

*Dr. Taft*—The subject of nomenclature is of the greatest interest and importance to our profession. Accuracy in conveying thought depends upon the words used; they should be definite and accurate, easily and readily apprehended. The use of language in the expression of thought is modified as the profession grows in knowledge of the language of science. Everything possible should be done for the attainment of the highest point. Discussions in associated bodies tend to the attainment of definite results. Teachers should lead in the right path. The effort of Dr. Kulp looks to the teachers in our profession. Any system generally adopted by them will come into general use in the future. The same system must be adopted, both in our institutes of learning and by those who write for the profession. The language used is often inappropriate, or conveys no definite idea. The plan proposed by Dr. Kulp is more definite and more universally applicable than anything heretofore proposed. It is sometimes feasible to use different words to avoid tautology. Masticating would be more definite than occluding. Occluding signifies "to come in contact," but all surfaces do not come in contact.

*Dr. W. N. Morrison*—Seven or eight years ago Drs. Judd and Dean produced a monograph on this subject, which was adopted in Chicago and St. Louis. The terms mesial and distal, when once defined and learned, looking to and looking from the median line, are simple and clear. Dr. Kulp's system is more complicated.

*Dr. Taft*—I am quite in favor of the terms used in the paper. Proximal is more easily understood; we should never go far beyond the apprehension of those who listen.

*Dr. Morgan*—The term occluding strikes me as incorrect when applied to the front teeth. When they are at rest they do not occlude. Cutting edges are not occluding surfaces.

*Dr. C. W. Spalding*—These points were very carefully discussed in the section. The terms grinding, masticating, and incising are each applicable in its own place, but occluding is more universally applicable; it is the only term that can be made to answer for all; it avoids multiplicity of words and is liable to fewer objections. Masticating applies to some, incising, to others; occluding will answer for all. Distal and mesial have no particular advantage; they are not plain English, and not understood by patients. Cuspo-lateral is plainer than disto-lateral. Mesial applies to surfaces looking to the centre; distal means more remote, but they are arbitrary in their application.

*Dr. Atkinson*—Whenever we attempt to name we must take nature's standard. We must avoid all complications. Occlude is the term, *par excellence*, to stand singly, signifying simply "bringing together." The terms we now use are picked up promiscuously, an *omnium gatherum* of all time.

*Dr. Taft*—In the comparison between the terms, occlusion is the incipient step in mastication.

*Dr. G. J. Friedrichs*—At every meeting of the Association, the last system of nomenclature offered is considered the best. I see no original outcome. At Niagara our report was almost snubbed. There is only one way of arriving at a uniform basis. Let a committee, either of College Faculties or from this Association, revise by sections this or some similar paper offered, and then let it be adopted; otherwise it is all balderdash.

*Dr. Sitherwood*—Said that whether this system or another was adopted, it was a matter of necessity that it be one which can be readily comprehended by both patients and students.

*Dr. Allport*—Considered the paper open to criticism. The molars were grinding teeth; the cutting edge of the incisors was not an occluding surface. Different terms must be used if we would have accuracy.

On motion, the subject was passed.

Section IV—Operative Dentistry, was called. The acting chairman, Dr. E. T. Darby, announced one paper, "The Painless Operation," by Dr. J. A. Robinson, of Jackson, Mich., and recommended several subjects for discussion, viz:

Bridgework; discussion to be opened by Dr. E. Parmly Brown.

The Herbst Method; discussion to be opened by Dr. M. L. Rhein.

The Perry Separators and Matrices; discussion to be opened by Dr. Darby.

Half Bicuspid Crowns; discussion to be opened by Dr. Noble.

Regulating Exhalation; discussion to be opened by Dr. Southwell.

Dr. W. C. Barrett read Dr. Robinson's paper, prefacing it by saying that "Uncle Jerry" as he is affectionately styled by all who know him well, being unable to attend, had submitted his paper to the section, which had accepted it, and designated him as the reader. Having had little opportunity to familiarize himself with the manuscript, he feared he might fail to do it justice. The following is a brief summary of the paper :

There is a universal desire to relieve or prevent pain. Tooth extraction is not desirable; it is not a blessing to the patient. We must be conservative rather than destructive. We encounter the greatest difficulty with friable teeth in anæmic conditions; palliative treatment sometimes is a necessity; an experience of fifty years has overcome many obstacles. The use of carbolized potash in preparing teeth for filling affords the most effectual relief. The brief pain occasioned by the application is nothing as compared to that of excavation, and we can give positive assurance that the latter will not hurt. If the pulp is exposed, first apply clear carbolic acid to cauterize it, then apply the remedy, and let it remain ten minutes; open with a large bur and the excavation will be painless. If there is any soreness, give the tooth rest; prevent occlusion with antagonizing teeth, which would keep up the irritation; shorten the cusps with corundum wheels. Gold is not always the best material for filling; amalgam is too often a failure; the textile foil prevents all shock from thermal changes. It has been said that it does not contain sufficient welding properties to hold a cap of gold, but I have never had a single failure with No. 6 foil welded to textile foil. Amalgam will be used for many years to come. Sinners are sometimes made better by good company; so is amalgam with textile foil; it makes a putty-like mass, composed of silver, tin, platinum, and gold. We must toil till we see the salvation of tooth-sinners; then, like Simeon of old, we can " depart in peace."

Dr. E. Parmly Brown, Flushing, L. I., opened the subject of Bridge-work. He said that, as many dentists had never seen any of this new work, he would only say that it was known at least two thousand years ago. The INDEPENDENT PRACTITIONER, some months since, gave an illustrated account of a genuine piece of bridge-work dug up in an ancient Etrurian burial place, which had doubtless been the property of an ancient princess. In laughing she must have shown a gold band, both inside and out, that completely exposed the deception. Recent improvements in modern dentistry have done away with the gold band, leaving, however, an unsightly gap between the teeth and the gums. As another step, far in advance, he now offered bridge-work (of which he exhibited specimens), with no metal visible, inside or out, and no open space for food to lodge in, but a close fit all around, sweet, and clean, and serviceable. He described a case where the right upper molar was healthy but pulpless, a mere shell, suitable for crowning; the second bicuspid was ulcerated and useless; the first bicuspid was sound. He extracted the abscessed root and inserted the new tooth in the wound, the porcelain tooth apparently growing out of the gum. The patient was so well pleased that he wanted the other side of the mouth treated in the same manner.

*Dr. Thomas*—Inquired how the strain to the teeth of attachment was obviated?

*Dr. Brown*—There is no difference in that regard between mine and any other method.

Dr. M. L. Rhein, of New York, opened the subject of the Herbst method (which was left unfinished at Saratoga). He said that during the past year he had modified his practice somewhat; that he does not now complete his work by the Herbst method. The special advantages offered are its superiority in adaptability of gold to the walls of the cavity without retaining pits or undercuts, the avoiding of nervous shock to the patient by the malleting, and the great saving of time over any other method. The larger and more extensive the approximal cavities in molars and bicuspids, the easier and more quickly in proportion can they be filled by the Herbst method, in comparison with other methods. The only disadvantage is that the gold is not so thoroughly condensed, not as much impacted as with the mallet, therefore he has modified his method and finishes the last one-third of the work with the electric mallet, thus obtaining contour, and thoroughly condensed masticating surface. Dr. Bodecker has done the same. Insert three-fourths, or seven-eighths of the filling up to the coronal surface, with the Herbst method, then compact with the mallet and get all the density required.

Dr. Herbst himself does not acknowledge that he cannot impact as much gold as by any other method, but others have not been able to do it. Cavities must all be reduced to simple cavities, applying the matrix for the missing wall in approximal cavities, a thin steel matrix held in place by heated shellac making the most difficult approximal cavities as simple as crown cavities in molars. Dr. Rhein exhibited specimens of work done by Dr. Herbst; a lateral in which two-thirds of the crown was built up in forty minutes, crown cavities in molars, the entire operation finished in from six to fifteen minutes, etc. Some of the work was done with tin-foil. Some of the teeth were split to show the perfect adaptation of the gold to the walls of the cavity. The gold used was a perfectly pure and very soft gold, prepared especially for this method, and used without annealing. The original is made by Wolrab, in Germany. S. S. White and Williams now offer American imitations, but which do not work like the original.

*Dr. Allport*—Does Dr. Herbst claim to weld tin ?

*Dr. Rhein*—The specimens have that appearance. The Wolrab gold works like rubbing the burnisher in a roll of butter.

Dr. Darby exhibited the Perry Separators, also various forms of matrices and appliances for holding the matrix in place. He said that Dr. Perry was one of the first to follow Dr. Jarvis in his method; the new separators are more out of the way, and control the pressure more evenly. He exhibited one adjusted to the incisors, separating the central from the lateral, with the matrix in position on the distal surface. Dr. Jack's method held the matrix in place with an orange-wood wedge. The present instrument is on the principle of that used for measuring the foot, or the monkey wrench, binding the matrix between the teeth. For separating molars and bicuspids it fits the teeth as the saddle does the horse. It does not interfere with the dam, does not incommode the patient, and is out of the way of the operator. Dr. Darby also exhibited Dr, Woodward's matrix, made of steel or phosphor-bronze, or brass, or copper. If of steel, the Seth Thomas watch spring makes the best matrix, and it may be cut with the plate shears. It is made with a lobe bent back upon the masticating surface of the adjoining tooth, which prevents it from slipping up to the gum. When the space is very narrow, a wedge can be used, shaped like a knife-blade. It is less liable to break than a wooden wedge, and keeps the matrix in place while holding the teeth a little apart. Phosphor-bronze is pliable and malleable, and is readily adapted to the space, forming a matrix-holder and separator combined.

Dr. Noble, of Washington, exhibited the half-bicuspid porcelain crown—a Howe crown, or tooth, with four pins, and a screw in root. This modification is adapted to the outer half of a bicuspid tooth, supplying an outer cusp to the natural inner cusp and root, and doing away with the labor of building up. It is faced with amalgam or cement filling, with a gold band embracing the cervical margin, and makes a strong and useful operation. Dr. Noble also showed Dr. Donalson's most recent invention, a machine for cutting barbs on all sides of his broaches, but not in a circle.

Dr. Southwell, of Milwaukee, exhibited an original invention for throwing the breath exhaled by patients away from the operator. It consists of a curved plate resting on the upper lip, to which is adjusted a shield which receives the air from the nostrils and throws it up and off to the right or the left, and away from the operator—especially adapted to operations on the upper teeth of a catarrhal patient. Dr. Southwell also read a paper, entitled "The Health of the Dentist," as affected by the close relation with patients, especially in the matter of inhaling the hot, moist breath, loaded with noxious exhalations of the lungs and nasal passages. He said that as a man cannot exist more than two minutes without air, while he may live eight or ten days without food, the necessity for air in proportion to food was as one to seven thousand, and yet how much more we think of the food we eat than of the air we breathe! The oxygen retained is equal to the carbonic acid gas exhaled; a slight percentage of impurity may be tolerated.

In inserting large fillings we are frequently so placed below and in front of the patient that the hot breath is directly within range of our lungs; we become light-headed and dizzy from inhaling carbonic acid gas, and nervous exhaustion results. In addition to this there is the constrained position, imperfect aeration of blood, vitiated atmosphere, underfeeding, etc. These things should have our earnest consideration, especially the importance of ventilation. With this little apparatus one fruitful source of ill-health will be obviated.

Dr. A. E. Matteson, of Chicago, exhibited an appliance which had been successfully used on a boy twelve years of age, who, in playing, had accidentally fallen and knocked out the central and lateral incisors. The process was broken off, hinging at a point near the foramen of the teeth; the teeth were knocked entirely out, and were picked up from the ground. The root canals were dressed with eucalyptus, and filled with gutta-percha. It was found very difficult to force them up into place. With the splint shown, which was cemented to the adjoining teeth, they were retained in place. The teeth were easily kept clean and sweet, and the boy never lost a meal. It proved simple and effective. He was assisted in the operation by Dr. Frank H. Gardiner. Dr. Matteson also exhibited his improved crowns. These are perfectly contoured shells, made of gold, lined with platina, giving the greatest amount of strength with least amount of material. They are readily thickened where required, as on the masticating surface, without melting the shell, which can be used as a crucible for melting the solder or gold scraps within the crown, thus securing correct articulation with the occluding teeth. Another great advantage is the ease with which a porcelain face can be inserted by cutting out the face of the shell, leaving a narrow band at the cervical margin, and inserting a platinum ring the width of the band, which rests on the end of the root, forming a shoulder, and which is soldered to the shell. For the porcelain front he uses a plain tooth, either with the pins or by grinding a dove-tailed slot, giving room for the screw-pin which is inserted in the root, the whole to be filled in with amalgam. The pin is of platinum wire, eighteen or twenty gauge, with screw-threads, the upper end bent so as to fall into the slot of the porcelain front when that is inserted. The crown is anchored to the root with amalgam, and the front cemented to the crown with oxy-phosphate. The root can be perfectly filled through the opening in the crown before the porcelain face is cemented on. A plaster cast of the adjoining teeth, and a cast of their antagonists, secure a perfect adaptation and articulation. The root must be in healthy condition, and the nerve canals filled at the apex. The end is to be ground off below the margin of the gum in front, and within an eighth of an inch of the gum on the lingual surface. The crowns, in assorted sizes, as also the gold and platina plate, and the dies and counter-dies for making the shells, can be obtained from the S. S. White Mfg. Co.

WEDNESDAY AFTERNOON SESSION

was devoted to Clinics, with Drs. Gardiner, of Chicago, Field, of Detroit, and Smith, of Minneapolis, as the managing committee.

Dr. Edmund Noyes, of Chicago, made a beautiful contour filling in a central incisor, using cohesive foil and the steel mallet.

Dr. E. Parmly Brown, using the electric mallet, made four very large contour fillings in the proximal and grinding surfaces of the first and second left superior bicuspids, and first molar.

Dr. T. T. Moore, of Columbia, S. C , made a very artistic contour filling in the anterior and palatine surfaces of the first superior right molar, also using the electric mallet.

Dr. I. C. St. John, of Minneapolis, made another gold contour filling in a proximal cavity of a central incisor, using a wooden mallet.

Dr. C. A. Timme, of Hoboken, N. J., made a large compound filling in a first left superior molar. He illustrated the Herbst method. The filling was dense and solid, but the proximal surface was not contoured, it having been previously filed flat.

Dr. M. L. Rhein, of New York, made a large compound filling in the posterior proximal and grinding surfaces of the left superior first bicuspid. He used Wolrab's German gold and the Herbst method for about two-thirds of the operation, lining the walls of the cavity at the margins with slightly annealed gold, and using the mallet to contour.

Dr. A. W. Harlan, of Chicago, treated two cases of pyorrhœa alveolaris, using Dr. Allport's new spring scalers.

Dr. J. J. R. Patrick, of Belleville, Ills., exhibited his device for striking up gold crowns, and Dr. Swasey, of Chicago, showed his apparatus for making corundum points, wheels, etc.

Dr. Matteson, of Chicago, inserted one of his porcelain front, gold and platinum crowns on a left superior lateral incisor root. The tooth presented a very natural appearance, and the operation promises to be a success.

The arrangements for the clinics were excellent, and the light all that could be desired. Chairs and other conveniences were placed in the hall, the seats being removed, and as the room was of sufficient size it gave excellent facilities for observing the different operations. At no meeting of the Association, at least for many years, have the arrangements for the clinics been so perfect, nor the attendance and interest so intense. Many new and interesting points were illustrated.

(TO BE CONTINUED.)

